# Researchers’ strategies to cope with the covid-19 impact on their activity

**DOI:** 10.1007/s12144-023-04601-5

**Published:** 2023-04-21

**Authors:** Anna Sala-Bubaré, Montserrat Castelló, Mariona Corcelles, Núria Suñé-Soler

**Affiliations:** Universitat Ramon LLull, Barcelona, Spain

**Keywords:** Researcher activity, Coping strategies, Covid-19, Well-being, Work and personal life balance, Research productivity

## Abstract

This study aims to characterize the strategies researchers used to cope with Covid-19 impact and to explore the relationship between those strategies, researchers’ characteristics and the pandemic impact in their lives. 721 researchers, proportionally distributed among three Spanish regions, answered an online survey on the pandemic impact on their activity. Scales referred to social support, productivity, research tasks, working conditions, and work and personal life balance. An open-ended section was included to collect the strategies they used to cope with the pandemic consequences. 1528 strategies were content analysed and categorised based on their purposes and related to the rest of the impact variables. Results show the predominance of some strategies for the whole sample both at the work level, such as organizing work duties and plans, and at the personal level, such as maintaining life-work balance and improving personal well-being. Results stress to what extent a strategic approach contributed to minimize contextual issues or constraints even in an extreme situation as the Covid-19 pandemic and lockdown. A non-strategic approach, consisting of just reacting emotionally or dropping research, was the less effective way to maintain interest in research, sustained work and productivity and to warrant work-life balance. Developing a strategic approach was easier for those without caring responsibilities and for men. Women in our study, especially with caring responsibilities, had reduced opportunities to continue with their careers during the pandemic. No evidence of institutional strategies supporting researchers to cope with the situation was found.

## Introduction

In the last years, academia has been the object of intense debates and claims for change, primarily due to the extreme increase of precarious positions, unequal opportunities, and overwork. Evidence is also accumulating about the mental health problems among junior researchers (Levecque et al., 2017) and more established academics (Kinman & Wray, 2013). Pressure to publish abundantly and quickly, engage in many and different types of research activities, and combine these with teaching and administrative issues is creating a culture of hyper-competitive levels of job security in which "systemic stress" is becoming normalized (Bekkouche et al., 2021).

The Covid-19 pandemic has added a whole new layer of complexity and uncertainty to an already tensioned profession (Denfeld, et al., 2020; Pinho-Gomes, et al., 2020; Watermeyer et al., 2020). Like many other professions (Watermeyer et al., 2020; Blahopoulou et al., 2022), researchers were asked overnight to work from home and required to continue working remotely, maintaining their usual activities (e.g., research, teaching, and administration) with no time to plan or adjust to the new situation, and sometimes even with little help or support from their institutions (Karademas & Thomadakis, 2021).

Two year into the pandemic, the accumulated evidence, mostly drawn from large survey reports and researchers’ personal accounts, shows that the pandemic has had a significant impact on the lives and work of researchers, with great differences in the extent to which individuals were able to continue their work (Kappel et al., 2021; Rijs & Fenter, 2020). Stress and burnout, changes in the role and responsibilities, increased workload, pressure in the already difficult life-work balance, and uncertainty regarding long-term decrease of research funding (Cameron et al., 2020; Kappel et al., 2021; Rijs & Fenter, 2020; Watermeyer et al., 2020) are the most common negative effects. Most of these studies report differential impact depending especially on the stage of the researcher career (De Gruyter, 2020; Gibson et al., 2020; Kappel et al., 2021).

Early career researchers faced specific barriers to keep doing research from home. They feel isolated from their colleagues and that they lack spaces for socialization and networking (Byrom, 2020; Cheng & Song, 2020; Levine & Rathmell, 2020; Wang & DeLaquil, 2020; Woolston, 2020). They experience decreased learning opportunities and less mentoring or supervision support (Cameron et al., 2020; De Gruyter, 2020; Eurodoc, 2020). Difficulties in accessing research resources, such as specialized hardware and software or labs facilities, and an adequate work environment were also highlighted (Byrom, 2020; Eurodoc, 2020). Evidence also shows that their psychological wellbeing was also significantly affected (Byrom, 2020; Denfeld, et al., 2020). Moreover, researchers at early career stages highlighted the negative impact that the pandemic had on their future and even present job prospects (Byrom, 2020; Denfeld et al., 2020; Eurodoc, 2020; Levine & Rathmell, 2020; Woolston, 2020). Other studies also indicate that productivity of early career researchers declined with the pandemic outbreak (Cui et al., 2020).

On the other hand, several studies show that the effects were worse for women, especially those at the beginning or middle of their careers (Ali et al., 2020; Andersen, et al., 2020; De Gruyter, 2020; Gewin, 2021), and those with children (Yildirim & Eslen‐Ziya, 2021), mostly due to increased difficulties in maintaining a life-work balance (Levine & Rathmell, 2020). Compared to men, women’s time devoted to research decreased more significantly, and consequently, the gender gap in research productivity was exacerbated (King & Frederickson, 2020; Pinho-Gomes, et al., 2020). Female researchers also experienced more stress, burnout, and loss of energy than male researchers (De Gruyter, 2020; Gewin, 2021). Similar results were found with university students (Almomani et al., 2021).

In sum, there is overwhelming evidence that the Covid-19 pandemic and the consequent contention measures have had a significant impact on researchers’ life and work around the world and across disciplines and stages. Although most of these studies and commentaries also devote many lines to provide recommendations to individuals and institutions to mitigate the negative effects, there is still little evidence of how researchers at different stages of their careers coped with the situation. To our knowledge, only two studies, Kappel et al. (2021) and Adarmouch et al. (2020), explored researchers’ coping strategies. In the first study, Kappel and colleagues (2021) asked 210 biology researchers across several countries to list the coping strategies that were effective in dealing with negative feelings or low mood related to the situation caused by the Covid-19 pandemic and consequent lockdown. Results showed that researchers mostly used exercise, outdoors activities and contact with friends and family, along with accepting the situation and practicing positive thinking, to cope with their negative feelings. In contrast, Adarmouch et al. (2020) asked to a group of 55 academic medical staff in Morocco about their strategies to maintain research activities, using a questionnaire with open-ended and multiple-choice questions. Their results show that the most frequent strategy was the use of communication technologies and remote work.

Our aim in this study was to further explore the coping strategies used by researchers, not only to manage specific challenges such as negative feelings or maintaining research, but more generally to cope with the diverse negative effects that the pandemic and consequent restrictions might have had in their lives and work. Ultimately, we aim to better understand how researchers adapt and cope with extremely demanding situations to contribute to facilitate early career researchers’ well-being and resilience when developing their careers. We know the situation created by the Covid-19 pandemic has impacted very differently in individuals depending on their gender, stage of career and family situation, among others. Thus, rather than asking how they dealt with specific challenges, we were interested in learning how these different groups cope with the changes and problems they had to face as a result of the Covid-19 pandemic. Specifically, the study aims:

- To characterize the strategies researchers used to cope with the impact of the Covid-19 pandemic.
- To explore the relationship between the strategies used, researchers’ characteristics and the impact the pandemic Covid-19 had in their lives.

## Method

### Participants

Participants in this study were 721 researchers working at universities in Catalonia (*n* = 438, 60.7%), the Valencian Community (*n* = 238, 33.0%) and the Balearic Islands (*n* = 45, 6.2%). The distribution among regions is proportional to the total numbers of researchers in each region. Of the 721 researchers, 408 were female (56.6%), 306 were male (42.4%), and two people were non-binary (0.3%), while five did not respond this question (0.7%). Women were slightly overrepresented with respect to the population of researchers in these regions.^1^ Their mean age was 45.4 years old (range 23–79).

Most participants were PhD holders (*n* = 509, 70.6%), some were doctoral researchers (*n* = 199, 27.6%) and the rest (*n* = 13, 1.8%) were technicians, research support staff or did not specify. Researchers were also distributed across disciplines: Social Sciences (28.7%), Sciences (24.9%), Humanities (18.8%), Health Sciences (15.9%) and Engineering and Architecture (11.7%). During the confinement, 47.1% of the participants had to take care of children, elderly, or ill relatives, and 21.8% experienced health issues.

### Instrument

Data were collected through the online survey “Researchers and Covid-19” aimed at exploring the effects of the Covid-19 pandemic on researchers at the individual, the institutional and disciplinary levels. The survey consisted of different scales, regarding impact of the Covid-19 pandemic on researchers’ work. Each scale contained 5-point Likert scale questions, from 1 = very positive impact to 5 = very negative impact. Scales items refer to 5 dimensions: social support, productivity, research tasks, working conditions, and balance between work and personal life (see Table 1 with survey items).

**Table 1:** Impact variables and related items

Impact variables	Items
Social support	Isolation
	Disconnection from other researchers
	Lack of guidance
Interest	Interest in my research/project(s)
	Appreciation of my work
Productivity	The rhythm of work
	Productivity
Research Tasks	Reading scientific publications
	Data collection and/or interventions
	Data analysis
	Dissemination and communication of results
	Preparation of applications for new projects or grants
Working conditions	Working or contractual conditions
	Future employment opportunities and conditions
Balance	Balance between research and other professional tasks (e.g. teaching, tutoring, etc.)
	Personal life balance

Likert scale 1 = very positive impact to 5 = very negative impact

The survey also included a section about the strategies they used to cope with the consequences of the Covid-19 pandemic, collected through an open-ended question (*What have you done to adjust to the changes you indicated?*) with three answer fields (*Strategy 1, Strategy 2, and Strategy 3*). The answer fields had no word-limit. Since the open-ended question about coping strategies was voluntary, respondents could leave them blank and continue answering the questionnaire.

The last section of survey asked for personal information, including whether they had caring responsibilities and health issues during the lockdown. The survey was available in Spanish and Catalan, and it required 10 to 15 min to complete.

### Data collection

Institutional e-mail addresses of researchers from all the universities in the three regions were collected from universities’ public websites. Between May and June 2020, potential participants were sent an email with basic information about the study and a link to the project’s website, where they could get further information about the aims and objectives, funding, risks, and advantages of participating, and had the opportunity to download the full questionnaire. Access to the survey was done through this website.^2^ The link to the questionnaire was not included in the email to increase transparency and ensure participants had complete information about the project characteristics and the ethical treatment of data before answering. At the beginning of the questionnaire, participants gave their consent to participate. The study and procedures were approved by the data protection officer of our institution.^3^ Among the 2149 answers received, 721 researchers (33.55%) responded the (voluntary) open-ended questions about coping strategies and were included in this study.

### Data analysis

A total of 1528 answers to the question *What have you done to adjust to the changes you indicated?* were collected. From the 721 participants, 45.4% (*n* = 327) provided three answers to the question, 28.7% of them provided two answers (*n* = 207) and 25.9% of participants provided one answer (*n* = 187).

Qualitative content analysis was used to scrutinize respondents’ answers about how they adjust to the changes. Emergent categories were established through a recursive process that involved the following steps: First, we read participants’ answers several times to note themes and patterns in the data. Second, we conducted a low-level inference coding process, using descriptive codes that paraphrased participants’ answers, which resulted in a large number of preliminary codes (*n* = 99). Third, we revised those codes to detect and address inconsistencies, mismatches, and overlaps. Fourth, the codes were grouped and classified into categories through discussion and reformulation of categories until agreement was reached about the definition of each category. In the few instances when respondents mentioned more than one theme in the same answer field, answers were double-coded (*n* = 27; 1.77%).

Two researchers, authors of this paper, independently coded a random selection of one third of responses and their analyses were compared to assess the reliability of the system of categories. Their agreement ranged from 79 to 88% among categories. Disagreements were discussed and resolved through consensus, resulting in fine-tuning of definitions and inclusion of some categories. The first author of the paper coded the rest of the data based on the final version of the coding scheme.

Further analysis was conducted to explore the relationship between the strategies used and researchers’ characteristics and the impact that the pandemic Covid-19 had in their lives. To this end, categories were organized into three groups based on their purposes: no strategy evidence, balance and well-being strategies and professional and research work strategies. Regarding researchers’ characteristics, we explored relationships between each category and participants’ gender (woman or man^4^), stage of career (pre-doctoral researchers or PhD holder^5^), caring responsibilities (yes/no) and health issues (yes/no) during the lockdown. Pearson’s chi-square test and corrected standardized residuals were used to analyze the association between pairs of variables.

The six impact variables were calculated using the mean of the items related to each variable (see Table 1). The relationship between the impact variables and participants’ strategies was calculated using the unpaired two-samples Wilcoxon test. For each strategy, two groups (use and no use of a given strategy) were compared in regards to each impact variable.

## Results

Ten thematic categories related to different ways to cope with the effects of the pandemic emerged from the analysis of the 1528 answers. As mentioned, categories were organized into three groups based on their purposes: no strategy evidence,^6^ balance and well-being strategies and professional and research work strategies. (see Table 2). The strategies most frequently mentioned by researchers were *organizing work duties and plans* (43.41%), *maintaining life-work balance* (25.80%), *adapting tools and research procedures to the new situation (23.86%)* and *improving personal well-being (20.53%).*

**Table 2:** Categories about the strategies to cope with the pandemic effects

Coping strategy	N people	% People
1. No strategy evidence		
1.1 No changes	37	5.13
1.2 Responding emotionally	53	7.35
2. Balance and well-being strategies		
2.1 Improving personal well-being	148	20.53
2.2 Sacrificing life-work balance	114	15.81
Maintaining life-work balance	186	25.80
3. Professional and Research work strategies		
3.1 Socializing and collaborating with colleagues	119	16.5
3.2 Organizing work duties and plans	313	43.41
3.3 Dropping research	63	8.74
3.4 Adapting tools and research procedures to the new situation	172	23.86
3.5 Focusing on specific research activities	107	14.84

### No strategy evidence

The first two categories of comments -*no changes* and *responding emotionally-* came from individuals (almost 13%) who either did not mention any strategic behaviour or refer to their emotional reaction as the unique way of dealing with the situation.

### No changes

Only 37 researchers (5.13%) were included in this category since they mentioned, more or less explicitly, not needing to do anything to adapt to the new situation, e.g., by saying the strategy was maintaining what they did before the pandemic. Comments like “I did not need to implement any strategy. I am used to it” (P1436), “Working as usual” (P1690) or “I was already teleworking before, it has not been a great change for me” (P1649) are prototypical of this category.

No statistically significant relationships were found between this category and participants’ sociodemographic characteristics.

Researchers who mentioned they did not need to change to cope with Covid-19 pandemic disruptions reported less negative impact on their interest regarding research (W = 9980, *p* < 0.05), their productivity (W = 18,241, *p* < 0.001), the events and deadlines (W = 17,729, *p* < 0.001), their research tasks (W = 16,884, *p* < 0.001), and their ability to balance research with their personal life and other professional tasks (W = 17,962, *p* < 0.001).

### Responding emotionally

This category represents those comments that imply individuals did not adjust to the situation but reacted to it. Thus, those responses did not include any action or strategy and only mention negative emotional reactions (‘getting angry with myself’, P1775), resignation (‘that’s how it is’, P4164), or explicitly referring to the lack of strategies (‘I could not cope, I am overwhelmed’, P3736). Emotional reactivity was mentioned by 53 researchers (7.35%).

No relationships were found between this category and participants’ sociodemographic characteristics. Participants who mentioned responding emotionally and showed reactivity as a response to the crisis were also more likely to experience greater negative impact in their productivity (W = 13,856, *p* < 0.01) and research tasks (W = 14,756, *p* < 0.05) and on their ability to balance research with their personal life and other professional tasks (W = 13,599, *p* < 0.01).

### Balance and well-being strategies

The second group refer to those strategies aimed at *improving personal wellbeing* and *sacrificing* or *maintaining* life *and work balance*, and were mentioned by more than two thirds of the sample.

### Improving personal well-being

148 individuals (20.53%) mentioned strategies and actions aimed at taking care of oneself, staying good and healthy, and handling the consequences of the pandemic better emotionally. Actions aimed at preserving or improving one’s health, with physical activity, rest and relaxation, or good eating habits, were very frequent, like in the following example: “Taking care of myself: yoga, healthy diet, sun exposure (terrasse)” (P3578). Other answers focused on psychological well-being. Researchers explained how they made efforts to stay positive and to take charge of their thoughts and attitudes: “Thinking more in the present without worrying too much about the future” (P911) and “Seeing it as a temporary situation” (P3326) are representative of this type of answers. Seeking support in personal networks (not professional networks or colleagues) were less frequent but also significant: “Keeping in touch with family and friends virtually” (P1544).

Women were more likely to mention this type of strategy than men (Χ^2^ = 5.30, df = 1, *p* < 0.05), as well as people who reported experiencing health problems in that period (Χ^2^ = 4.55, df = 1, *p* < 0.05). No relationships were found between this type of strategy and the impact reported in participants’ research.

### Sacrificing life-work balance

This category, which was mentioned by 114 researchers (15.81%), included actions that jeopardized individuals’ wellbeing and healthy balance between their personal life and work in order to keep up with work. More specifically, researchers mentioned working more (“Working more and faster”, P1959; “Working non-stop”, P2937) and at ill-timed hours (“Working until sunrise”, P2082). Many mentioned sacrificing their sleep, and some even explicitly said they renounced spending time with their families: “I had to abandon my family life to maintain to keep up” (P4178).

Women were much more likely to mention this type of coping actions than men (Χ^2^ = 5.72, df = 1, *p* < 0.05). Likewise, PhD holders were more likely to mention them than doctoral researchers (Χ^2^ = 23.56, df = 1, *p* < 0.001). Individuals who had caring responsibilities during the confinement were much more likely to mention sacrificing life-work balance than those who did not caring responsibilities (Χ^2^ = 22.96, df = 1, *p* < 0.001). Researchers who worked in a team were also more likely to mention these coping action than those working mostly individually or both individually and collaboratively (Χ^2^ = 15.32, df = 1, *p* < 0.001).

Researchers who used this strategy were more likely to report greater negative impact on their productivity (W = 29,055, *p* < 0.01), research tasks (W = 20,238, *p* < 0.001) and their ability to balance research with their personal life and other professional tasks (W = 13,599, *p* < 0.01).

### Maintaining life-work balance

This category, mentioned by 186 researchers (25.80%), includes actions oriented towards finding a balance between personal life and work at home, either by organizing schedules and routines taking into account wellbeing and family time (“finishing the workday at a previously stipulated hour that allows me to enjoy time with my family/partner or do exercise without being exhausted at the end of the day”, P3427) or by creating a working space at home (“organizing my home in separate spaces for work and rest”, P3889). Some include family organization to define working spaces and schedules (“pact with the members of the family to organize space and time of each person”, P3590).

Using this type of strategies did not significantly relate to any sociodemographic characteristics. Individuals who mentioned these strategies reported negative impact on their ability to balance research with their personal life and other professional tasks, but to a lower extend than those people who did not mention strategies related to maintaining life-work balance (W = 55,157, *p* < 0.05).

### Professional and research work strategies

Finally, almost all the participants referred to strategies directly related to their research work such as *socializing and collaborating with colleagues*, *organizing work duties and plans*, *adapting tools and research procedures*, *focusing on specific research activities* and *dropping research*.

### Socializing and collaborating with colleagues

Comments included within this category refer to actions and strategies related to teamwork and research teams, either with a focus on professional socialization or on work, and were mentioned by 119 researchers (16.50%). Many researchers mentioned staying in touch with their colleagues, to support each other, socialize or stay in touch (“creating a Slack space open to all researchers in the area to exchange experiences and provide support to each other”, P2585). Less frequent comments involved organization of teamwork (“dividing the project and achievement of objectives among researchers”, P2937).

Using this type of strategies did not significantly relate to any sociodemographic characteristics or perceived impact.

### Organizing work duties and plans

This category was the most frequent, with almost half of participants (*n* = 313, 43.41%) mentioning at least one strategy related to it. Actions and strategies included in this category were related to organizing work, without any mention to balance with personal life, either by setting or modifying work schedules and routines (“establishing a strict working schedule”, P889), planning time and tasks (“try to establish a schedule for each task”, P948) or establishing priorities (“prioritizing objectives and goals”, P2874). Less frequent answers in this category were related to setting self-imposed objectives or deadlines, presumably to maintain motivation and continue working (“setting short- and long-term objectives”, P2176).

Doctoral researchers were much more likely to mention this kind of strategy than PhD holders (Χ^2^ = 35.88, df = 1, *p* < 0.001), as well as those individuals who had no caring responsibilities during the pandemic (Χ^2^ = 6.14, df = 1, *p* < 0.05). Researchers who mentioned these strategies reported negative impact on their ability to balance research with their personal life and other professional tasks, but to a lower extend than those people who did not mention organization strategies (W = 72,873, *p* < 0.01).

### Adapting tools and research procedures to the new situation

This category, mentioned by 172 researchers (23.86%), includes actions or strategies aimed at adapting on-going research, projects, and work activities to the new needs or to the consequences of the pandemic on their work. Many researchers mentioned the need to learn and start using new tools to work from home (“making efforts to master the new meeting software and looking for new software for conferences and lessons”, P3787). Other researchers commented on the need to adapt the objectives, procedures, or chronogram of their research projects due to the current situation (“adapting the research I was doing to the context of Covid-19 (methodologically and theoretically)”, P4193). Less frequent answers mentioned teleworking (either alone or in combination with in-person work) as a strategy to cope with the situation (“partial telework (computer tasks, data analysis and writing scientific articles) and the rest [of the time] in the lab for the experimental tasks”, P3364).

PhD holders were more likely to mention adapting their research to the new situation than pre-doctoral researchers (Χ^2^ = 3.85, df = 1, *p* < 0.05). Individuals who mentioned these strategies reported negative impact on their productivity, working conditions and life-work balance, but to a lower extend than those people who did not mention strategies related to adapting tools and research procedures (productivity: W = 53,721, *p* < 0.01;, working conditions: W = 51,576, *p* < 0.05; and balance: W = 52,147, *p* < 0.05).

### Focusing on specific research activities

Finally, this category includes actions or strategies related to continuing doing research and working and was mentioned by 107 researchers (14.84%). Most of the answers were related to focusing on one task or activity as their strategy (“taking the time to make progress in computer tasks (courses, data analysis, writing)”, P1416; or “writing papers with data obtained before Covid”, P1977). Less frequent topics were finishing pending tasks or projects (“intensifying work, as I applied for two research projects and one has been granted”, P3501). Across these topics, many researchers mentioned they had more time to do research as they did not have to commute (“using the time I devoted to commute to do research” P1716).

Researchers working in a team were less likely to mention this strategy than those working mostly individually or both individually and, in a team (Χ^2^ = 9.96, df = 1, *p* < 0.01).

Researchers who mentioned using this type of strategies reported negative impact on their productivity, research tasks and ability to find life-work balance, but to a lesser extend than those who did not report this type of strategies productivity: W = 39,281, *p* < 0.001; research tasks: W = 37,908, *p* < 0.05; and balance: W = 37,178, *p* < 0.05).

### Dropping research

63 researchers (8.74%) mentioned renouncing to do research, completely or partially. Some researchers mentioned adjusting expectations, given that they could not keep up with all the research work and tasks as before (“Lowering the expectations regarding research productivity and assuming that it is not possible to continue working as nothing was happening”, P3955), while others said what they did was rejecting projects (“renouncing projects due to impossibility to assume more work”, P2054), prioritizing other activities over research (“I drastically reduced the research workload due to the sudden demands on teaching”, P1163), or giving up research completely (“stop doing research. I haven’t written a single line despite having pre-covid commitments”, P1924).

Researchers with caring responsibilities were more likely to mention this type of coping action (Χ^2^ = 11.54, df = 1, *p* < 0.001).

Researchers who mentioned dropping their research differed greatly from those individuals who did not mention it. Specifically, they reported greater negative impact on their interest for research (W = 23,771, *p* < 0.05), productivity (W = 11,188, *p* < 0.001), events and deadlines (W = 13,522, *p* < 0.001), research tasks (W = 13,178, *p* < 0.001) and ability to balance their research with their personal life and other professional duties (W = 12,570, *p* < 0.001).

## Discussion

The study aimed to explore the coping strategies used by researchers at different stages of their careers during the first Covid-19 lockdown, a stressful and unexpected situation in which research demands did not stop but practices were altered (Woolston, 2020). Specifically, we looked for not only how researchers managed specific challenges such as negative feelings or maintaining productivity, but more generally coped with the diverse effects that the pandemic and consequent restrictions might have had in their lives and work.

Results show the predominance of some strategies for the whole sample both at the work level, such as organizing work duties and plans, and at the personal level, such as maintaining life-work balance and improving personal well-being. Perhaps given the prevalence of these strategies, we did not find differences in the impact related to their use. However, we can hypothesize that strategies to maintain life-work balance, to preserve well-being and to organise work in some cases might have had a preventive effect, while for other people it might have been the only way to mitigate the disruptions in their personal and professional life. It is remarkable that the few participants who did not mention any strategy experienced no negative impact on their productivity, research activity or work-life balance, which point towards the existence of a group of researchers who did not have to change substantially their research activity despite the pandemic situation. However, the majority experienced personal and professional concerns and challenges and needed to put into play a variety of strategies to cope with them. Results stress to what extent a strategic approach contributed to minimize contextual issues or constraints even in an extreme situation as the Covid-19 pandemic and lockdown. A non-strategic approach, consisting of just reacting emotionally or dropping research, was the less effective way not only to maintain interest in research, sustained work and productivity but also to warrant work-life balance, which in many cases was the researchers' final purpose. Developing a strategic approach was easier for those without caring responsibilities and for men. These individuals were able to combine strategies aiming at maintaining work-life balance with others related to organizing professional work and particularly focusing or prioritizing some specific tasks above others. This ability to decide how to adapt and prioritize depending on the context dynamics is at the core of strategic researcher activity and accounts for relevant dimensions of researcher career development such as research writing and dissemination, mobility, networking or team management and leadership (Castelló et al, 2015). As in previous studies, women in our study, and especially those with caring responsibilities, had reduced opportunities to continue with their careers during the pandemic (Andersen et al., 2020; De Gruyter, 2020; Gewin, 2021; King & Frederickson, 2020; Pinho-Gomes, et al., 2020).

On the other hand, despite the evidence of the negative effects of the pandemic in career prospects (Woolston, 2020), strategies specifically targeting these consequences were not found in our study. Although many reported advancing their research, all strategies focused on dealing with present issues, not planning their career. The moment when we collected data, during the first total lockdown, might explain this result. During the initial pandemic outbreak, uncertainty was high but Higher Education was not affected by mass redundancies, which could explain why participants did not exhibit strategies related to career prospects. Career planning and other long-term strategies might appear later, after one year and a half of the pandemic.

All the reported strategies were at the individual level, seeking for personal adjustments. In some cases, strategies were related to connecting with colleagues and their research team. However, we did not found evidence of institutional strategies supporting researchers to cope with the situation. Given the size of the sample and its representativity across career stages, we can assume that a sufficient number of respondents had institutional responsabilities, so the lack of institutional strategies mentioned by participants is surprising.

The study is not without limitations. First, the transversal design adopted, exclusively based on self-report answers, does not allow us to understand to what extent the strategies used effectively helped researchers to deal with the pandemic impact and minimize the challenges and negative effects through time. There is a risk of circular explanation since we only look at one moment in a period where adaptation and change were probably key.

However, the study provides evidence of some strategies having a preventive role, while others much more reactive seem to be less adaptive in maintaining both emotional stability and research work progress. It is well known researchers must be resilient and usually work under pressure and in difficult situations (Bekkouche et al., 2021; Kinman & Wray, 2013); Such evidence has practical implications for PhD students and early career researchers training. Knowing how, when and why a set of strategies help researchers deal with these situations might help us to facilitate the knowledge and management of a set of strategies for them to cope with tensions intentionally, difficulties and distress throughout researcher career development. Moreover, such strategies’ relationships with wellbeing and productivity might reduce doctoral drop-out (Castelló et al., 2017), enhance early career wellbeing and better contribute to doctoral training and researchers’ career development.

## Data Availability

The datasets generated during and/or analysed during the current study are available in the https://osf.io/ repository, https://osf.io/d8khx/

## Footnotes

1 According to the Integrated System of University Information of the Spanish Ministry of Universities (http://estadisticas.mecd.gob.es/EducaJaxiPx/Tabla.htm?path=/Universitaria/Personal/EPU19/PU//l0/&file=PU0201.px), in 2019 these three regions employed a total of 47,102 researchers (including graduate researchers and research assistants): 27,486 researchers were employed at a Catalan university (58.4%), 17,653 researchers were working at a Valencian university (37.5%), and 1,909 researchers were employed at a Balearian universities (4.1%). 43.4% of these researchers were female.

2 https:// [information removed for blind peer review].

3 Study design, procedure and tools were approved by the ethics committee of the [information removed for blind peer review].

4 Participants were given four options to identify their gender: ‘woman’, ‘man’, ‘my gender is best described as: (please complete)’, and ‘I do not wish to answer’. However, the frequency of answers in the last two categories was very low (0.3% and 0.7% respectively) and thus were not considered in the analysis of the gender differences. While this may have been useful for methodological purposes, we are aware this classification does not capture the complexity of gender identities.

5 The third category, ‘other researchers’ was excluded from further analysis due to the low prevalence in the sample (1.8%).

6 Since we adhere to the existing conceptualization of strategies as intentional actions or procedures directed towards a goal (González-Ocampo & Castelló, 2019; Schmeck, 2013), reacting emotionally or doing nothing cannot be conceptualized as a strategy unless there is further evidence of intentions or reasons behind this behavior. However, considering the nature of our data, with "no strategy evidence" we do not claim that participants did not use any strategy, but rather that their answer does not provide evidence of whether they did.
